# An Innovative Non-Linear Prediction Model for Clinical Benefit in Women with Newly Diagnosed Breast Cancer Using Baseline FDG-PET/CT and Clinical Data

**DOI:** 10.3390/cancers15225476

**Published:** 2023-11-20

**Authors:** Ken Kudura, Nando Ritz, Arnoud J. Templeton, Tim Kutzker, Martin H. K. Hoffmann, Kwadwo Antwi, Daniel R. Zwahlen, Michael C. Kreissl, Robert Foerster

**Affiliations:** 1 Department of Nuclear Medicine, Sankt Clara Hospital, 4058 Basel, Switzerland; 2 Department of Radiology, Sankt Clara Hospital, 4058 Basel, Switzerland; 3 Sankt Clara Research, 4002 Basel, Switzerland; 4 Division of Nuclear Medicine, Department of Radiology and Nuclear Medicine, University Hospital Magdeburg, 39120 Magdeburg, Germany; 5 Faculty of Medicine, University of Basel, 4001 Basel, Switzerland; 6 Faculty of Applied Statistics, Humboldt University, 10117 Berlin, Germany; 7 Department of Radiooncology, Cantonal Hospital Winterthur, 8400 Winterthur, Switzerland

**Keywords:** PET/CT, FDG-PET/CT, breast cancer, clinical benefit, prediction model, generalized additive model

## Abstract

**Simple Summary:**

This study aimed to develop an innovative non-linear statistical model to predict clinical benefit in women with newly diagnosed breast cancer. A logistic generalized additive model was chosen as an innovative statistical approach, as opposed to conventional techniques. Clinical data, primary tumor (PT) features on baseline [^18^F]-fluorodeoxyglucose positron emission tomography/computed tomography (FDG-PET/CT), and molecular subtype were considered for the purpose of the investigations. In this retrospective study of 70 women, higher primary tumor volume and metabolic parameters significantly compromised clinical benefit. A multivariate model for clinical benefit, incorporating age, body mass index, T, M, PT total lesion glycolysis, and PT volume, demonstrated excellent accuracy across the molecular subtypes. Our results emphasized the pivotal role of baseline FDG-PET/CT in predicting treatment outcomes. However, careful consideration is warranted when choosing the methodological approach for treatment outcome prediction, as non-linear influences of predictive biomarkers on clinical benefit were unveiled.

**Abstract:**

**Objectives:** We aimed to develop a novel non-linear statistical model integrating primary tumor features on baseline [^18^F]-fluorodeoxyglucose positron emission tomography/computed tomography (FDG-PET/CT), molecular subtype, and clinical data for treatment benefit prediction in women with newly diagnosed breast cancer using innovative statistical techniques, as opposed to conventional methodological approaches. **Methods:** In this single-center retrospective study, we conducted a comprehensive assessment of women newly diagnosed with breast cancer who had undergone a FDG-PET/CT scan for staging prior to treatment. Primary tumor (PT) volume, maximum and mean standardized uptake value (SUVmax and SUVmean), metabolic tumor volume (MTV), and total lesion glycolysis (TLG) were measured on PET/CT. Clinical data including clinical staging (TNM) but also PT anatomical site, histology, receptor status, proliferation index, and molecular subtype were obtained from the medical records. Overall survival (OS), progression-free survival (PFS), and clinical benefit (CB) were assessed as endpoints. A logistic generalized additive model was chosen as the statistical approach to assess the impact of all listed variables on CB. **Results:** 70 women with newly diagnosed breast cancer (mean age 63.3 ± 15.4 years) were included. The most common location of breast cancer was the upper outer quadrant (40.0%) in the left breast (52.9%). An invasive ductal adenocarcinoma (88.6%) with a high tumor proliferation index (mean ki-67 expression 35.1 ± 24.5%) and molecular subtype B (51.4%) was by far the most detected breast tumor. Most PTs displayed on hybrid imaging a greater volume (12.8 ± 30.4 cm^3^) with hypermetabolism (mean ± SD of PT maximum SUVmax, SUVmean, MTV, and TLG, respectively: 8.1 ± 7.2, 4.9 ± 4.4, 12.7 ± 30.4, and 47.4 ± 80.2). Higher PT volume (*p* < 0.01), SUVmax (*p* = 0.04), SUVmean (*p* = 0.03), and MTV (<0.01) significantly compromised CB. A considerable majority of patients survived throughout this period (92.8%), while five women died (7.2%). In fact, the OS was 31.7 ± 14.2 months and PFS was 30.2 ± 14.1 months. A multivariate prediction model for CB with excellent accuracy could be developed using age, body mass index (BMI), T, M, PT TLG, and PT volume as predictive parameters. PT volume and PT TLG demonstrated a significant influence on CB in lower ranges; however, beyond a specific cutoff value (respectively, 29.52 cm^3^ for PT volume and 161.95 cm^3^ for PT TLG), their impact on CB only reached negligible levels. Ultimately, the absence of distant metastasis M displayed a strong positive impact on CB far ahead of the tumor size T (standardized average estimate 0.88 vs. 0.4). **Conclusions:** Our results emphasized the pivotal role played by FDG-PET/CT prior to treatment in forecasting treatment outcomes in women newly diagnosed with breast cancer. Nevertheless, careful consideration is required when selecting the methodological approach, as our innovative statistical techniques unveiled non-linear influences of predictive biomarkers on treatment benefit, highlighting also the importance of early breast cancer diagnosis.

## 1. Introduction

With an estimated 2.5 million new cases occurring in 2020, breast cancer has become globally the most commonly diagnosed cancer, surpassing lung, colorectal, and prostate cancers [1]. Among women, breast cancer is the leading cause of cancer-related mortality, accountable for 14.7% of cancer-related deaths in women. However, striking disparities in breast cancer incidence and mortality, influenced by socioeconomic factors and lifestyle choices, have been observed, both across countries and within nations [2,3,4,5]. In western countries, breast cancer mortality rates have witnessed a decline over recent decades, even as incidence rates have risen [6]. This reduction in mortality has been attributed to noteworthy advancements in early diagnostic techniques, such as mammography, ultrasound, and breast magnetic resonance imaging (MRI), alongside hybrid imaging, and the introduction of more effective targeted systemic treatments [7].

An accurate assessment of disease extent is required for an effective management of breast cancer [8]. The [^18^F]-fluorodeoxyglucose positron emission tomography/computed tomography (FDG-PET/CT) has emerged as a valuable tool for identifying regional lymph node involvement or distant metastasis but also for an appropriate follow-up, particularly in cases of locally advanced breast cancer. Hence, FDG-PET/CT has been recommended in numerous studies recently published for staging from stage IIB onwards (depending on the histological subtype) and for assessing treatment response. Additionally, it may contribute significantly to cases of suspected tumor recurrence [6,8,9,10,11,12,13,14,15,16,17].

Recent investigations have spotlighted the utility of baseline FDG-PET/CT for predicting outcomes in melanoma and non-small-cell lung cancer (NSCLC) patients [18,19,20]. In the context of breast cancer, there has also been a rising interest in recent literature in using FDG-PET/CT as hybrid imaging to predict response to treatment, particularly in the context of neoadjuvant systemic therapy [21,22,23,24,25,26,27,28,29,30,31,32,33,34]. Despite a significant heterogeneity of variables defined as outcomes, most of the published models predicting treatment response in breast cancer have predominantly relied on linear statistical approaches, such as Cox proportional hazards analysis. This approach often fails to adequately consider the possibility of non-linear influences of the predictive variables [28,30,31,33,35,36].

Hence, we opted for the use of cutting-edge statistical techniques, as opposed to conventional methodological approaches, to develop a novel non-linear statistical model integrating primary tumor features on baseline FDG-PET/CT, molecular subtype, and clinical data for treatment benefit prediction in women with newly diagnosed breast cancer.

## 2. Materials and Methods

### 2.1. Patient Selection

In the context of this single-center retrospective study, we conducted a comprehensive assessment of female patients newly diagnosed with breast cancer between 1 January 2017 and 31 December 2021 at the Sankt Clara Hospital in Basel, Switzerland. Inclusion criteria encompassed adult female patients with histopathologically confirmed breast cancer who had undergone FDG-PET/CT scan for staging prior to any local or systemic treatment and had provided their informed consent for the use of their data for research purposes.

### 2.2. Data Collection

The following clinical data were obtained from medical records, which included decision reports from the interdisciplinary tumor board:
**Data Category****Specifics**Patient DemographicsAge, GenderAnthropometric DataHeight, Weight, and Body Mass Index (BMI)Primary Tumor CharacteristicsAnatomical site and histologyReceptor StatusEstrogen receptor (ER) expression, Progesterone receptor (PR) expression, and Human Epidermal Growth Factor Receptor-2 (Her-2) expression/overexpressionTumor Proliferation Indexki-67 expressionMolecular SubtypeLuminal A, Luminal B, HER-2 enriched, or triple negativeClinical StagingTNM (8th edition American Joint Committee on Cancer AJCC)

Three endpoints were assessed on the same date, 10 February 2023:
**Endpoint****Definition**Overall survival (OS)The time from the date of diagnosis to death or the last follow-upProgression-free survival (PFS)The time from the date of diagnosis to disease progressionClinical benefit (CB)No death and no disease progression from the date of diagnosis to the last follow-up

### 2.3. FDG-PET/CT Acquisition

Two PET/CT scanners were used in clinical routine within the considered time window: a PET/40-detector CT scanner (Biograph 40 TruePoint True V PET/CT, Siemens Healthcare, Erlangen, Germany) from 1 January 2017 to July 2020, followed by a PET/64-detector CT scanner (Discovery Molecular Insights (DMI) PET/CT, General Electrics (GE) Healthcare (Waukesha, WI, USA) from August 2020 to 31 December 2021.

All PET/CT data used in this single-center retrospective study were acquired in clinical routine and conducted in accordance with the department’s standard protocol. The procedure entailed the intravenous administration of ^18^F-FDG after a minimum four-hour fasting period. One hour later, a diagnostic CT scan was performed with dedicated chest acquisition for attenuation correction and morphological characterization of lesions, extending from the skull to the thighs, with the patient in supine position. Subsequent to the CT scan, a static 3D PET acquisition was performed with the patient in identical position. PET images were subsequently reconstructed using an ordered subset expectation maximization (OSEM), with a threshold set at 42% of the maximum standardized uptake value (SUVmax) and a time-of-flight (TOF) correction. Iodinated contrast medium was used in absence of renal impairment, allergy, or other contraindications. Details of the injected activity and dose length product (DLP) were documented.

Both PET/CT scanners were regularly calibrated. An additional statistical analysis was performed to investigate whether the use of two distinct PET/CT scanners influenced the measurement of semiquantitative parameters.

### 2.4. Primary Tumor (PT) Segmentation on FDG-PET/CT

All of the enrolled PTs were retrospectively meticulously delineated prior to any treatment. The delineation process was performed on co-registered PET- and CT-images, facilitated by an advanced workstation (AW) version 4.7 provided by GE Healthcare, employing a manual three-dimensional contouring tool. Ensuring consistency, the delineation process involved a careful manual alignment of tumor borders on both PET and CT images before proceeding with any measurements. Subsequently, the following primary tumor characteristics were recorded:

- Morphological features: volume, morphology (solid, inflammatory), and margin (sharp, irregular, spiculated)
- Metabolic features: SUVmax, SUVmean, metabolic tumor volume (MTV), and total lesion glycolysis (TLG)

### 2.5. Statistical Analysis

For the purpose of descriptive statistical analyses, the study cohort was dichotomized into two groups: patients with CB versus patients without CB.

Continuous variables were summarized using mean values and their respective standard deviations (SD), while categorical variables were characterized through frequency distributions. A *t*-test was performed to compare the means of continuous variables between women with CB versus women with no CB. Additionally, a chi-squared test was applied to assess the distribution of categorical variables across these groups. The criterion for statistical significance was set at *p* < 0.05.

Moreover, to explore the potential impact of utilizing two different PET/CT scanners on the semiquantitative parameters measured, an analysis of variance (ANOVA) was conducted.

Subsequently, a logistic generalized additive model (GAM) was chosen as the statistical approach to assess the impact of all listed variables on the CB of treatment. This modeling choice was made because of its efficacy in capturing complex, non-linear relationships inherent in the data [36,37,38]. The determination of model parameters was conducted through a meticulous stratified bootstrap procedure, ensuring that the statistical properties of the stratified subsets aligned harmoniously with those of the original dataset. This innovative statistical approach involved averaging parameter estimates across an extensive set of simulations, surpassing 10,000 iterations. In doing so, the model’s parameters could be refined and the model’s goodness of fit was estimated with great scrutiny. The performance of the generated logistic GAM was evaluated first for the entire cohort and afterwards with regards to the molecular subtypes using receiver operating characteristic (ROC) curves. Lastly, Kaplan–Meier survival curves were generated by molecular subtype to evaluate OS and PFS.

All statistical analyses were performed using R (version 4.1.1).

## 3. Results

### 3.1. Patient Selection

**Figure 1:**
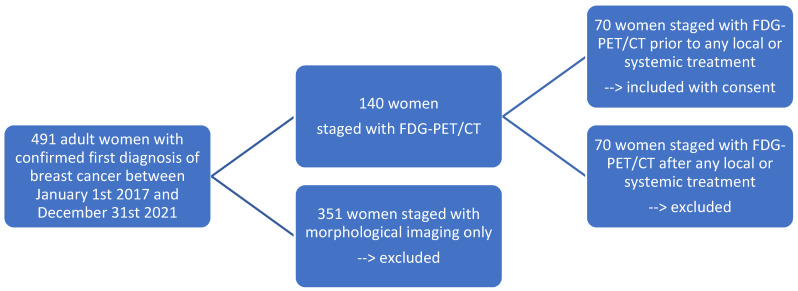
Flow chart of patient selection (N = 70) according to our inclusion and exclusion criteria.

Out of a total of 491 adult women who were newly diagnosed with breast cancer between 1 January 2017 and 31 December 2021, 140 women underwent FDG-PET/CT for staging purposes. Half of these individuals had the imaging prior to any form of local or systemic treatment, while the other half had already received a form of treatment. Ultimately, 70 adult women, satisfying our inclusion criteria and having provided their informed consent, were included in the study (Figure 1).

### 3.2. Descriptive Statistics

Seventy women with a first diagnosis of breast cancer were included in this study, with an average age of 63.3 ± 15.4 years and an average BMI of 26.5 ± 5.7 kg/m^2^. These individuals underwent a FDG-PET/CT scan for staging, with an average blood glucose level of 5.7 ± 0.9 mmol/L, an average injected activity of 304.8 ± 102.1 MBq, and an average DLP of 833.6 ± 388.5 mGy·cm (See Table 1).

The most prevalent site of breast cancer occurrence was the upper outer quadrant (40.0%), predominantly located in the left breast (52.9%). In the majority of cases, the diagnosis revealed an invasive ductal adenocarcinoma (88.6%), characterized by a high tumor proliferation index with a mean ki-67 index of 35.1 ± 24.5%. The predominant molecular subtype identified was luminal subtype B (51.4%) (See Table 1 and Table 2).

FDG-PET/CT was primarily performed at stage T2 (48.6%), N1 (48.6%), and M0 (82.3%), which corresponds to stage IIB (8th edition of the AJCC). On hybrid imaging, most PTs showed solid morphology (94.3%), irregular margins (78.6%), larger volumes (mean volume of 12.8 ± 30.4 cm^3^), and notable hypermetabolism (mean ± SD of PT SUVmax, SUVmean, MTV, and TLG, respectively: 8.1 ± 7.2, 4.9 ± 4.4, 12.7 ± 30.4, and 47.4 ± 80.2) (See Table 1 and Table 2).

The average follow-up duration for patients during this study was 34.4 ± 12.7 months. The vast majority of patients survived throughout this period (92.8%), while five women died (7.2%). The mean overall survival was 31.7 ± 14.2 months and the mean progression-free survival was 30.2 ± 14.1 months (Table 1).

Hybrid imaging revealed significant disparities between patients experiencing CB and those without CB. These differences encompassed the presence of distant metastases (*p* < 0.01) an PT volume (*p* < 0.01), as well as PT metabolic features, respectively, PT SUVmax (*p* = 0.04), SUVmean (*p* = 0.03), and MTV (*p* < 0.01). Additionally, substantial distinctions were observed in PFS (*p* < 0.01) and mortality (*p* < 0.01) between these two groups (See Table 1 and Table 2).

Notably, our analysis yielded no significant disparities in the measured semiquantitative parameters across both PET/CT scanners, as determined through ANOVA.

### 3.3. Prediction Model Development

**Figure 2:**
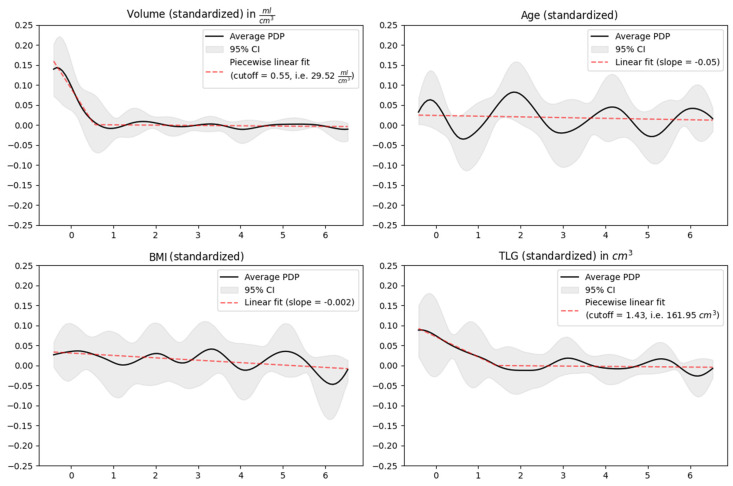
Standardized and harmonized continuous parameters of the prediction model for CB. (**In the first row**): PT volume and patient age; (**in the second row**): patient BMI and PT TLG.

The progressive inclusion of variables in the final model followed a structured five-step protocol. First of all, correlation matrices were generated and deployed to identify and separate strongly correlated variables, encompassing both continuous and categorical ones. Subsequently, all continuous features underwent a standardization process to achieve uniformity in their scales (Figure 2). The third pivotal step involved the use of a stratified bootstrap strategy to split the dataset into distinct test and training subsets, facilitating the meticulous choice of optimal hyperparameters for the number of splines and lambda through an exhaustive grid search. Subsequently, as the fourth step, the model was estimated within each bootstrap iteration (in total over 10,000 iterations), taking into account the hyperparameters derived in the previous step. Finally, in the fifth and last step, all parameters were harmoniously averaged to consolidate our findings.

This innovative statistical approach resulted in a multivariate prediction model for the CB of treatment with the following standardized and harmoniously averaged predictive parameters: age, BMI, T, M, PT TLG, and PT volume.

PT volume was the strongest continuous predictive biomarker, followed by PT TLG. Our results captured a non-linear influence of continuous predictive biomarkers on CB. In fact, PT volume and PT TLG demonstrated a significant influence on CB in lower ranges. However, beyond a specific cutoff value (respectively, 29.52 cm^3^ for PT volume and 161.95 cm^3^ for PT TLG), their impact on CB approached negligible levels. When considered independently, the remaining continuous predictive parameters (respectively, age and BMI) exhibited a marginal effect on CB (Figure 2).

**Figure 3:**
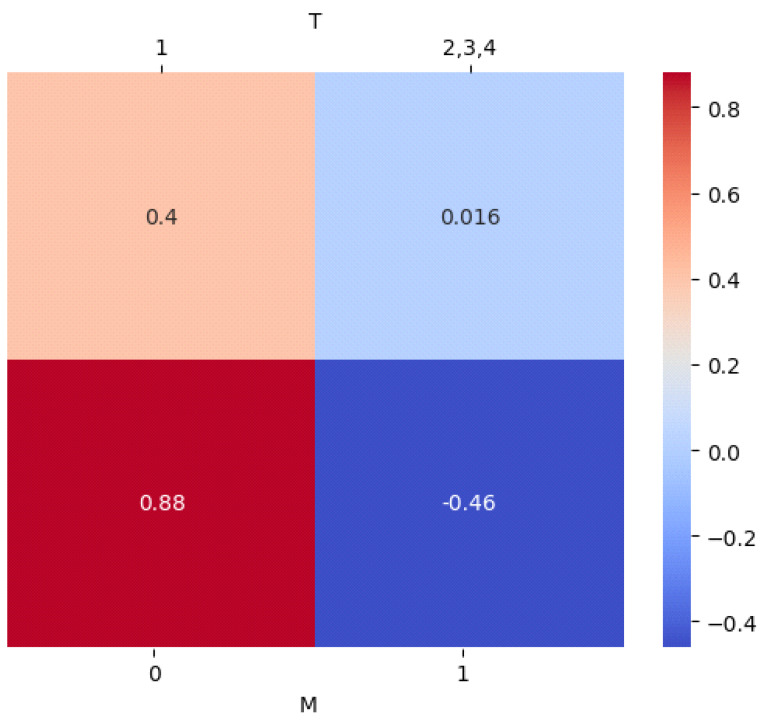
Standardized average estimates for categorical parameters of the prediction model for CB. First, both categorical variables were dichotomized into two events: (**In the first row**): T1 vs. T > 1; (**in the second row**): M0 vs. M1.

Among the categorical predictive biomarkers, the absence of distant metastasis on hybrid imaging displayed the strongest positive impact on CB far ahead of the tumor size T (standardized average estimate for 0.88 vs. 0.4) (Figure 3).

On the *X*-axis is the standardized parameter (for a better comparison among the parameters) and on the *Y*-axis is the influence of the considered parameter on CB. Values around 0.00 on the *Y*-axis indicate a negligible influence of the parameter on CB. PT volume showed the strongest influence on CB, followed by PT TLG in lower ranges up to a cutoff value of 29.52 cm^3^ and 161.95 cm^3^, respectively. Above this value, the respective impact on CB approached negligible levels. When considered independently, the remaining continuous parameters of the prediction model (e.g., age and BMI) exhibited a marginal effect on CB.

Afterwards, over 10,000 stratified bootstrapping procedures were carried out to assess the standardized average influence of each state of the categorical parameters on CB: T1 = 0.4 vs. T > 1 = 0.016; M0 = 0.88 vs. M1 = −0.46.

### 3.4. Performance of the Generated Prediction Model

#### 3.4.1. For the Entire Cohort (N = 70)

**Figure 4:**
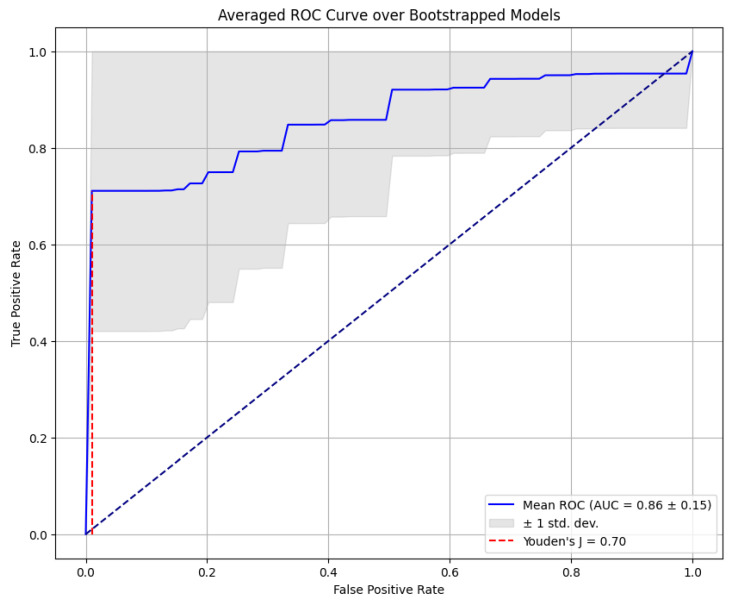
Receiver operating characteristics curve of the generated multivariate prediction model for CB in women with first diagnosis of breast cancer.

In order to validate the accuracy of the developed multivariate prediction model for CB, its goodness of fit was evaluated for the entire group of women newly diagnosed with breast cancer. This evaluation involved the construction of a ROC curve, which captured an excellent predictive power across the entire cohort (area under the curve AUC 0.86 ± 0.15; Youden index 0.70) (Figure 4).

#### 3.4.2. According to the Molecular Subgroups

**Figure 5:**
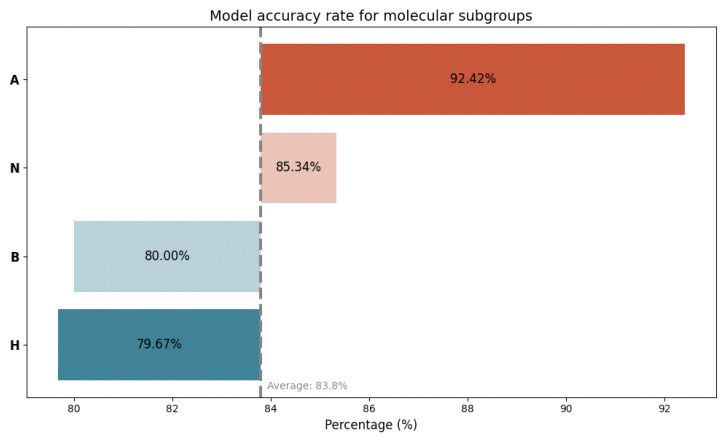
Accuracy rate of the generated multivariate prediction model for CB, taking into account the molecular subtype. A = luminal A breast cancer; N = triple-negative breast cancer; B = luminal B breast cancer; H = Her2-amplified breast cancer.

Furthermore, we scrutinized the performance of the generated prediction model in both high-risk and low-risk scenarios, taking into account the molecular subtype. An excellent average level of predictive accuracy was achieved across all subgroups, with the best performance in women with luminal A breast cancer, interestingly followed by women with triple-negative breast cancer (Figure 5).

### 3.5. Survival Analysis

**Figure 6:**
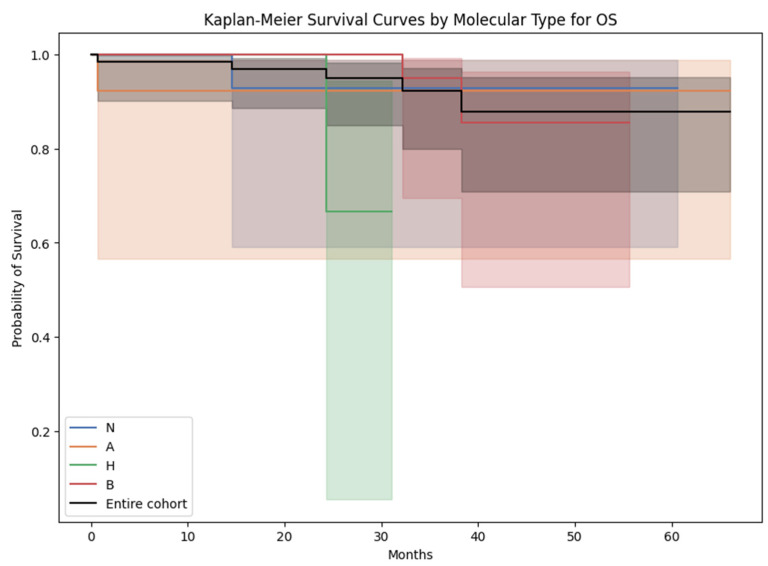
Kaplan–Meier survival curves for overall survival across the molecular subtypes. N = triple-negative breast cancer; A = luminal A breast cancer; H = Her2-amplified breast cancer; B = luminal B breast cancer.

The mean ± SD of OS (in months) in women diagnosed with luminal A, luminal B, Her2-amplified, and triple-negative breast cancer was, respectively, 34.8 ± 16.6, 31.2 ± 13.8, 21.6 ± 9.1, and 34.3 ± 14.0. Interestingly, upon conducting a chi-squared test with Bonferroni correction, no statistically significant disparities in OS were discerned across these molecular subtypes (Figure 6).

**Figure 7:**
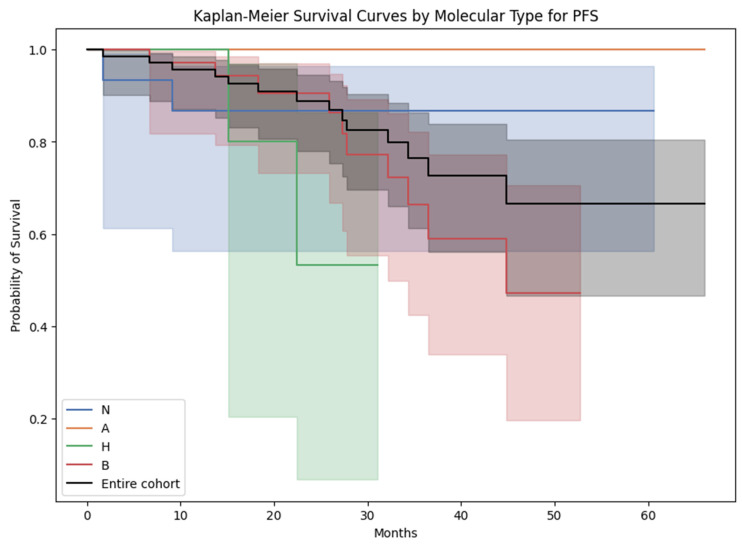
Kaplan–Meier survival curves for progression-free survival across the molecular subtypes. N = triple-negative breast cancer; A = luminal A breast cancer; H = Her2-amplified breast cancer; B = luminal B breast cancer.

The mean ± SD of PFS (in months) in women diagnosed with luminal A, luminal B, Her2-amplified, and triple-negative breast cancer was 34.8 ± 16.6, 26.7 ± 12.6, 20.5 ± 9.3, and 33.5 ± 15.4. Notably, no statistically significant differences were observed in PFS among these molecular subtypes, as determined by a chi-squared test with Bonferroni correction (Figure 7).

It is important to observe that OS and PFS were not employed as endpoints for the developed prediction model, attributable to constraints stemming from the sample size and a comparatively low mortality rate. The survival analysis presented above using OS and PFS as endpoints aimed primarily to assess potential differences across molecular subgroups with regards to their survival but did not yield statistically significant outcomes (Figure 6 and Figure 7).

## 4. Discussion

We aimed to use cutting-edge statistical techniques, as opposed to conventional methodological approaches, to develop a novel non-linear statistical model integrating primary tumor features on baseline FDG-PET/CT, molecular subtype, and clinical data for treatment benefit prediction in women with newly diagnosed breast cancer.

The descriptive statistical analysis of our cohort spotlighted at least three significant insights, with some results aligning with established knowledge, while others warrant further discussion.

Among the seventy predominantly older and overweighted Caucasian women included in our study, invasive ductal adenocarcinoma emerged as the predominant breast tumor histology. These tumors exhibited a high tumor proliferation index, were predominantly classified as luminal molecular subtype B, and were most commonly located in the upper outer quadrant of the left breast. These observations were consistent with existing well-established knowledge in the field. Notably, the prevalence of invasive ductal adenocarcinoma across our cohort reflected its global status as the most prevalent form of invasive breast cancer. Moreover, our findings resonated with recent research highlighting the significant role of age, diet, and weight in the genesis and progression of breast tumors [12,39].

Secondly, the utilization of FDG-PET/CT for staging, primarily at stage IIB, aligned with international recommendations for breast cancer staging [6,8,9,10,11,12,13,14,15,16].

Thirdly, significant differences were captured during pre-treatment FDG-PET/CT in women with no CB, compared to women manifesting CB. In fact, higher metabolism and volume of the primary tumor, as much as the presence of distant metastases on hybrid imaging prior to any treatment, significantly compromised CB and were associated with a higher mortality rate and shorter PFS in our cohort. The heightened metabolic activity observed in the delineated primary tumors throughout our entire cohort was intriguing, especially in light of the predominance of luminal tumors among the women included in our study. Previous research has often indicated that tumoral uptake tends to be the highest in Her-2 overexpressing tumors, followed by triple-negative tumors, with luminal breast cancers generally exhibiting lower levels of uptake [10,11,40,41,42]. However, the elevated metabolic activity we observed across the cohort may be attributed to the relatively high proliferation index, particularly predominantly in cases of invasive ductal adenocarcinoma. This aligned with recent investigations, such as the study by Groheux et al. which reported that histological subtype and proliferation index exert an influence on tumoral uptake. Specifically, invasive ductal adenocarcinoma and high proliferative breast cancers were associated with greater tumoral uptake [11]. Overall, higher metabolism of breast cancers on hybrid imaging prior to any treatment compromised the benefit of treatment, in accordance with existing knowledge [43,44,45,46,47].

There has been a growing inclination in recent literature toward the use of FDG-PET/CT as a non-invasive hybrid imaging tool for forecasting treatment response in women with newly diagnosed breast cancer, especially in the context of neoadjuvant systemic therapy [21,22,23,24,25,26,27,28,29,30,31,32,33,34]. A vast majority of the existing models for treatment response prediction in breast cancer have traditionally leaned toward linear statistical methodologies, notably the Cox proportional hazards analysis. This conventional approach tends to overlook the possibility of non-linear influences of the predictive variables [28,30,31,33,35,36].

A logistic generalized additive model was chosen as an innovative statistical approach to assess the impact of all listed variables on CB of treatment in our cohort. This modeling choice was rooted in its multidisciplinary approach (with regards to the data incorporated into the model) but above all, its efficacy in capturing complex, non-linear relationships inherent in the data [37,38]. Consequently, we developed a multivariate prediction model incorporating age, BMI, T, M, PT TLG, and PT volume as predictive biomarkers, with CB as the primary endpoint. Several notable strengths of our developed prediction model warrant attention.

First and foremost, our results unveiled a non-linear relationship between continuous predictive biomarkers and CB. Specifically, PT volume and PT TLG exhibited a substantial influence on CB within lower ranges. However, beyond specific cutoff values (29.52 cm^3^ for PT volume and 161.95 cm^3^ for PT TLG), their impact on CB diminished considerably. This observation bears significant clinical relevance, emphasizing the predictive potential of PT morphology and metabolic volume and the importance of early diagnosis across various histological and molecular subtypes. Notably, breast cancers detected at an early stage with lower volume and metabolic activity may not only be associated with improved treatment benefits but also reduced healthcare costs [7,48].

Secondly, the predictive performance of our multivariate model was outstanding, not only across the entire cohort but also in high-risk subgroups, such as Her-2 overexpressing and triple-negative breast cancers [9,12,15,32].

Lastly, while all variables detailed in Section 2 were initially considered in our modeling approach, the most influential predictive biomarkers for clinical benefit in our final prediction model were derived from FDG-PET/CT scans conducted prior to any treatment. This underscored a pivotal role played by baseline FDG-PET/CT in forecasting treatment outcomes for women newly diagnosed with breast cancer. In 2019, Phung et al. conducted a comprehensive review comprising 54 model development studies in the context of breast cancer. Most of these studies adopted a retrospective study design and had sample sizes ranging from 70 to 433 patients [30]. However, the attempt to conduct a more detailed comparison of our model with those previously reported in their review encountered significant limitations. These limitations were primarily due to substantial heterogeneity across the reviewed studies, particularly concerning the definition of outcome variables and patient selection criteria (e.g., inclusion of patients before any local or systemic treatment, variations in histological or molecular subtypes included). Additionally, while the majority of the reviewed studies predominantly employed Cox proportional hazards regression for model development, we chose to employ innovative statistical techniques, such as generalized additive models (GAM).

Finally, the present study had inherent limitations, primarily related to its retrospective nature and relatively modest sample size. However, these characteristics were consistent with numerous model development studies in this field [30]. Despite these limitations, our study has yielded valuable and statistically robust insights.

Furthermore, the use of two distinct PET/CT scanners during the follow-up period did not have a significant impact on the measured semiquantitative parameters.

Lastly, no external validation of the presented prediction model could be conducted given the single-center retrospective study design initially chosen. Further, multicenter investigations involving larger cohorts might be needed as external validation of our results.

## 5. Conclusions

Our results emphasized the pivotal role played by FDG-PET/CT prior to treatment in forecasting treatment outcomes in women newly diagnosed with breast cancer. Nevertheless, careful consideration is required when selecting the methodological approach, as our innovative statistical techniques unveiled non-linear influences of predictive biomarkers on treatment benefit, highlighting also the importance of early breast cancer diagnosis.

## Figures and Tables

**Table 1:** Descriptive statistics of continuous variables for the entire cohort (N = 70); patients with CB (55) vs. no CB (15).

N	All		CB		No CB		*p*-Value
	70		55		15		
	Mean	SD	Mean	SD	Mean	SD	
Age (years)	63.3	15.4	63.5	15.3	62.8	16.3	0.88
BMI (kg/m^2^)	26.5	5.7	26.2	5.7	27.7	5.7	0.36
Blood glucose (mmol/L)	5.7	0.9	5.7	0.9	6.0	1.1	0.33
Injected activity (MBq)	304.8	102.1	300.1	98.2	323.1	118.1	0.46
Total DLP (mGy·cm)	833.6	388.5	826.6	390.4	859.4	393.9	0.77
PT Volume	12.8	30.4	7.5	11.1	32.1	59.7	<0.01
PT SUVmax	8.1	7.2	9.0	7.8	4.7	2.4	0.04
PT SUVmean	4.9	4.4	5.5	4.7	2.8	1.5	0.03
PT MTV	12.7	30.4	7.5	10.9	32.0	60.0	<0.01
PT TLG	47.4	80.2	44.3	77.3	58.9	92.1	0.54
Ki-67 expression (%)	35.1	24.5	35.5	23.9	34.0	27.4	0.84
Observation time (months)	34.4	12.7	33.7	12.7	36.9	12.8	0.39
OS (months)	31.7	14.2	32.5	13.4	28.8	17.2	0.37
PFS (months)	30.2	14.1	32.5	13.4	21.4	13.4	<0.01

**Table 2:** Descriptive statistics of categorical variables for the entire cohort (N = 70); patients with CB (55) vs. no CB (15).

Clinical Data	All	CB	No CB	*p*-Value
Anatomical site				1.00
1 = right	33 (47.1%)	26 (42.3%)	7 (46.7%)	
2 = left	37 (52.9%)	29 (52.7%)	8 (53.3%)	
Quadrant				0.86
1 = central position	7 (10.0%)	5 (9.1%)	2 (13.3%)	
2 = upper inner quadrant	11 (15.7%)	9 (16.4%)	2 (13.3%)	
3 = lower inner quadrant	7 (10.0%)	6 (10.9%)	1 (6.8%)	
4 = upper outer quadrant	28 (40.0%)	23 (41.8%)	5 (33.3%)	
5 = lower outer quadrant	16 (22.9%)	11 (20.0%)	5 (33.3%)	
9 = not further described	1 (1.4%)	1 (1.8%)	0	
Histology PT				0.25
1 = invasive ductal adenocarcinoma	62 (88.6%)	50 (91.0%)	12 (80.0%)	
2 = invasive lobular adenocarcinoma	5 (7.1%)	3 (5.4%)	2 (13.3%)	
3 = invasive papillary adenocarcinoma	1 (1.4%)	0 (0.0%)	1 (6.7%)	
4 = mucinous carcinoma	1 (1.4%)	1 (1.8%)	0 (0.0%)	
5 = apocrine carcinoma	1 (1.4%)	1 (1.8%)	0 (0.0%)	
Molecular subtype PT				0.33
A = Luminal A	13 (18.6%)	12 (21.8%)	1 (6.7%)	
B = Luminal B	36 (51.4%)	26 (47.3%)	10 (66.7%)	
H = Her-2 enriched	6 (8.6%)	4 (7.3%)	2 (13.3%)	
N = triple negative	15 (21.4%)	13 (23.6%)	2 (13.3%)	
T				0.17
1	21 (30.0%)	19 (34.5%)	2 (13.3%)	
2	34 (48.6%)	27 (49.1%)	7 (46.7%)	
3	2 (2.9%)	1 (1.8%)	1 (6.7%)	
4	13 (18.6%)	8 (14.6%)	5 (33.3%)	
N				0.54
0	19 (27.1%)	17 (30.8%)	2 (13.3%)	
1	34 (48.6%)	26 (47.3%)	8 (53.3%)	
2	6 (8.6%)	4 (7.3%)	2 (13.3%)	
3	11 (15.7%)	8 (14.6%)	3 (20.1%)	
M				<0.01
0	58 (82.3%)	51 (92.7%)	7 (46.7%)	
1	12 (17.1%)	4 (7.3%)	8 (53.3%)	
Hybrid Imaging	All	CB	No CB	*p*-Value
Margin PT				0.78
1 = sharp	10 (14.3%)	7 (12.7%)	3 (20.0%)	
2 = irregular	55 (78.6%)	44 (80.0%)	11 (73.3%)	
3 = spiculated	5 (7.1%)	4 (7.3%)	1 (6.7%)	
Morphology PT				0.42
1 = solid	66 (94.3%)	53 (96.4%)	13 (86.7%)	
2 = inflammatory	4 (5.7%)	2 (3.6%)	2 (13.3%)	
Death				<0.01
0 = no	65 (92.8%)	55 (100.0%)	10 (66.7%)	
1 = yes	5 (7.2%)	0 (0.0%)	5 (33.3%)	

## Data Availability

The data presented in this study are available on request from the corresponding author.

## Abbreviations

FDG-PET/CT	[^18^F]-fluorodeoxyglucose positron emission tomography/computed tomography
PT	Primary Tumor
SUVmax	Maximum Standardized Uptake Value
SUVmean	Mean Standardized Uptake Value
MTV	Metabolic Tumor Volume
TLG	Total Lesion Glycolysis
OS	Overall Survival
PFS	Progression-Free Survival
CB	Clinical Benefit
BMI	Body Mass Index
MRI	Magnetic Resonance Imaging
NSCLC	Non-Small-Cell Lung Cancer
ER	Estrogen Receptor
PR	Progesterone Receptor
Her-2	Human Epidermal Growth Factor Receptor-2
AJCC	American Joint Committee on Cancer
DMI	Discovery Molecular Insights
GE	General Electrics
OSEM	Ordered Subset Expectation Maximization
TOF	Time-of-Flight
DLP	Dose Length Product
AW	Advanced Workstation
SD	Standard Deviation
ANOVA	Analysis of Variance
GAM	Generalized Additive Model
ROC	Receiver Operating Characteristic
AUC	Area Under the Curve
